# Accreditation of new technologies for predicting intramuscular fat percentage: Combining Bayesian models and industry rules for transparent decisions

**DOI:** 10.1371/journal.pone.0314714

**Published:** 2025-03-24

**Authors:** Graham E. Gardner, Clair L. Alston-Knox

**Affiliations:** 1 School of Veterinary and Life Sciences Murdoch University, Murdoch, Western Australia, Australia; 2 Predictive Analytics Group, Melbourne, Victoria, Australia; National University of Computer and Emerging Sciences,PAKISTAN

## Abstract

The experiment evaluated a method for statistically assessing the accuracy of technologies that measure intramuscular fat percentage (IMF%), enabling referencing against accreditation accuracy thresholds. To compare this method to the existing rules-based industry standard we simulated data for 4 separate devices that predicted IMF% across a range between 0.5–9.5% for sheep meat. These devices were simulated to reflect increasingly inaccurate predictions, and the two methods for statistically assessing accuracy were then applied. We found that for the technology which only just meets the accreditation accuracy standards, as few as 25 samples were required within each quarter of the IMF% range to achieve 80% likelihood of passing accreditation. In contrast, using the rules based approach at least 200 samples were required within each quarter of the IMF% range, and this increased the likelihood of passing to only 50%. This method has been developed into an on-line analysis App, which commercial users can freely access to test the accuracy of their technologies.

## Introduction

**Fig 1:**
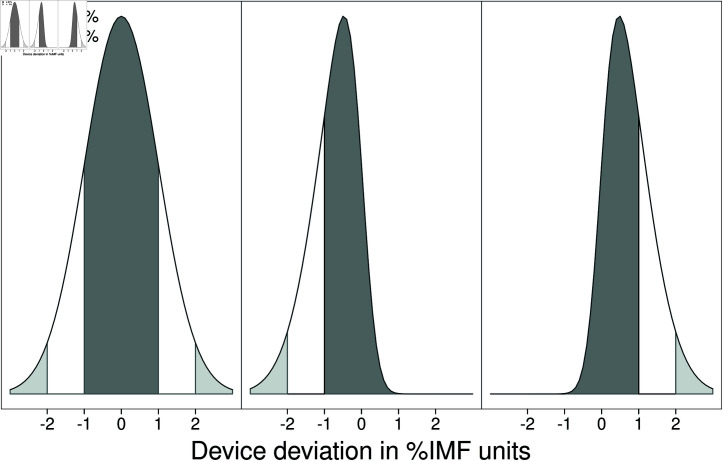
Potential distributions of IMF% prediction errors that meet accreditation standard. The industry standard for accreditation does not require device deviations to be symmetrical. The minimum requirement is 67% (or greater) of samples within 1 IMF% of the laboratory estimate and less than 5% exceeding 2 IMF%. These 3 examples are equally acceptable in terms of device accreditation.

Intramuscular fat (IMF%) is a key determinant of eating quality in lamb [1], and therefore the Australian meat industry uses it as an input into the new Meat Standards Australia cuts-based eating quality prediction model [2]. The measurement of IMF% is undertaken by a laboratory-based Soxhlet fat extraction method [3] which cannot be used in abattoirs as it is destructive and has a slow processing speed. To aid in implementation, technologies are being developed that have sensors capable of predicting this trait and are able to keep pace with lamb-abattoir processing speeds. However, these technologies must first be accredited for use by the Australian Meat Industry Language and Standards Committee which specifies minimum accuracy standards for measuring IMF% in lamb. The standards that the committee have mandated to test accuracy is that 67% of these estimates must fall within  ± 1 IMF% of their laboratory value, while 95% of predictions must fall within  ± 2 IMF%. Technologies seeking accreditation must predict the IMF% in 20 carcasses within every IMF% increment for which they are seeking accreditation. There is no requirement for the device deviations from laboratory estimates to be symmetric (i.e. normally distributed errors) so long as they still meet the minimum accuracy standards, as demonstrated in Fig 1. These same accuracy standards are also applied to repeatability testing which must be demonstrated both within a device across 3 measurements, and between 3 separate devices.

Adherence to these accuracy standards is currently assessed by determining the number of values that exceed the accuracy thresholds and representing them as a percentage of the total population sampled. There are several limitations to this rules-based approach.

Firstly, this does not assess whether the accuracy standards are maintained across the range of values for which accreditation is sought. Indeed, if the accuracy of the technology was best at lower IMF% values then accreditation could be achieved by ensuring that a substantial portion of the accreditation data is captured at low IMF% levels while still maintaining the minimum 20 samples required at the higher IMF% levels.

The second limitation is associated with the potential to incorrectly “fail” the accreditation attempt of a device due to random error in sampling a population. For example, if a device has an error of prediction that is Normally distributed, with an acceptable accuracy, say a N(0,1), then it should be accredited based on industry determined standards. These rules are based on the envisaged performance of the device on the population of carcasses it will measure in the future. As we are taking a sample of that population, then, *on average*, this device will pass the industry set rules. However, as this is a sample, each sample will generally fall above/below the average 50% of the time for each of the two rules, giving the device a slightly less than 50% chance of passing accreditation.

Additionally, the calculation of percentages changes with sample size, in that a requirement of 67% may in fact require much larger proportions. This is due to the rounding of samples, particularly small samples of less than 100. These rules are also subject to failure through the random presence of outliers. This can be overcome through increasing the sample size, thus minimising the impact of outliers, and maximising the impact of observations that fall within 1IMF%. However, increasing sample size represents a major cost-burden on the sheep industry.

Shortcomings in the rules based approach, particularly for devices that fall just within the required industry standards, are the basis for considering an alternative method for assessing the accuracy of a technology against the IMF% accuracy standards. This alternative method must a) still test the accuracy thresholds for a device to pass accreditation, b) be robust to non-symmetric data and c) be readily understood by industry partners.

An approach to device accreditation that meets all of these requirements utilises a Bayesian linear regression approach to parameter estimation based on the utility of the posterior distribution. Estimating parameters using posterior distributions is computationally convenient, as we wish to focus on the predictive distribution of future observations (currently unobserved) to estimate the expected performance of the device if many samples were obtained. The predictive posterior is easily formed as it is a simple sum of the jointly estimated posterior for the model parameters and variance. Additionally, in a Bayesian framework, specific sample sizes to obtain the required power to make statements of significance are not a primary consideration. Rather, as sample size increases, the estimates of the posterior distribution for the coefficients and the error variance will become more precise.

In this paper we have undertaken a simulation study using the existing accreditation standards for measuring IMF% in sheep carcasses, aiming to demonstrate the effect of sample size on the probability of a technology passing accreditation. We then test an alternative modelling technique based upon Bayesian linear regression, and demonstrate its comparative efficiency through a further simulation study that assesses the effect of sample size upon the probability of a technology passing accreditation. We hypothesise that the Bayesian linear regression approach will require less samples to arrive at robust estimates of accreditation accuracy, representing a large economic benefit to the industry.

## Materials and methods

### Simulated data

In order to demonstrate the comparative effectiveness of the rules based approach versus a Bayesian regression modelling proposal for testing accreditation accuracy, data sets were simulated for 4 separate devices that predicted IMF% across a range between 0.5–9.5% for sheep meat. Data were simulated for devices assuming the following error distributions:

- Assume 1 SD = 1 IMF%.
- Simulate values within the thresholds {0.5–1.5, 1.5–2.5, 2.5–3.5, ⋯ , 8.5–9.5} using a Uniform distribution. Simulate the maximum sample size for IMF.
- Simulate the errors (maximum sample size) of the device/s as follows:
  – Device A: This device is better minimum requirement for accreditation:  ⋯ 
  – Device B: This device just meets minimum requirement for accreditation:  ⋯ 
  – Device C: this device is marginally worse than the minimum requirement for accreditation:  ⋯ 
  – Device D: This device is slightly worse again in terms of minimum requirements for accreditation:  ⋯ 
- For each sample size required (*n*), use the first *n* samples of the complete simulation.

### Rules based approach

The current criteria for device accreditation is that, overall, the device estimates of IMF% must be at least of the standard;

1. 67% of device estimates are within  ±  1 IMF% compared to laboratory estimates
2. 95% of device estimates are within  ±  2 IMF% compared to laboratory estimates.

If the difference between the laboratory and the device estimates were symmetric, this would equate to differences between the device and the laboratory being distributed as N(0, 1.03^2^). The rationale behind these rules is based in the ideal that a device which has the largest proportion of disparity between a laboratory estimate and device estimate being within  ±  1 IMF%, and very few disparities outside  ±  2 IMF% would be acceptable in terms of grading meat cuts for consumer use. The general impetus of these rules was that a device with errors (compared to a laboratory) that were N(0,1^2^) would be the maximum acceptable deviation in estimates of IMF% that the industry would accept.

The problem with “bright line rules” is that they are formed based on the concept of a complete population, ignoring the expected uncertainty associated with sampling. By definition, a sample from a N(0,1^2^) will, on average, have 68% of values within  ±  1IMF%. However, exactly 68% is unlikely in a sample, and as such, the outcomes will be distributed symmetrically around 68%, with roughly 50% of samples meeting the requirement to exceed 68%.

**Fig 2:**
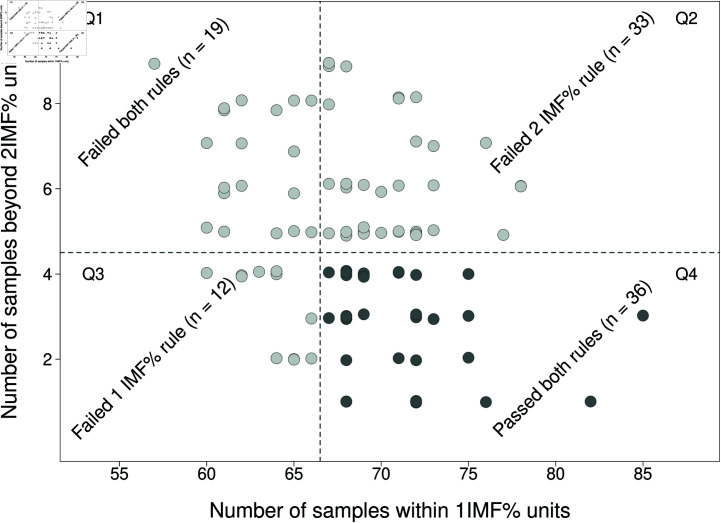
Simulations of sample size 100 from a N(0,1). If the device just meets the requirements to pass accreditation then of the 100 experiments, 36 of them passed both rules. A further 33 passed the 1IMF% rule only, 12 passed the 2IMF% rule only and 19 failed both rules. Such a device will not necessarily pass accreditation based on a single sample set. There are 100 simulated datasets in this figure.

This concept is demonstrated in Fig 2 which shows an example of 100 simulated samples from a N(0,1) each with a sample size of 100. From the industry perspective, a device with errors from this distribution is suitable for accreditation. The requirements to pass accreditation are slightly reduced by introducing the rule that more than 67% of samples must be within  ±  1 IMF %. We can clearly see that the number of simulations that contain samples with 67% or more within  ±  1 IMF% is slightly more than half, with 69 of the 100 simulations passing the 1 IMF% rule (quadrants Q2 & Q4). In this simulation study, 48 of the 100 simulations passed the requirement that less than 5% of samples will be more than 2 IMF % units from the true value (quadrants Q3 & Q4), which is close to 50% , as would be expected when using a sample of the population. However, the sample needs to simultaneously pass the rules associated with both the 1 & 2 IMF % thresholds (quadrant Q4). In this sample there were 36 simulations that passed both rules. Any device that is seeking accreditation could be represented as a single simulation, hence, if the device is just within satisfactory range for accreditation, in this simulation study, it would have a probability of only 36% of passing the rules based requirement.

Fig 2 clearly illustrates that the bright line rules have a tendency to incorrectly reject devices, which, based purely on their statistical precision and accuracy, should be accredited. For example, the 97th simulation (located in quadrant Q3) was rejected as it had only 66% of samples within 1 IMF% units. However, the next largest sample was a deviation of 1.01 IMF% and it had only 2 deviations greater than 2 IMF%. From a management perspective, it may be tempting for companies applying for accreditation to just keep adding individual samples until they have a set of data that meet the rules based requirements. This is active p-hacking, akin to optional stopping [4] and will do nothing to improve either the fairness or robustness of the accreditation scheme. Alternatively, there may be subjective rounding applied to the device data, allowing data on the boundary of 1 IMF% or 2 IMF% to be allocated in a more favourable region, which in this case is quadrant Q4.

**Fig 3:**
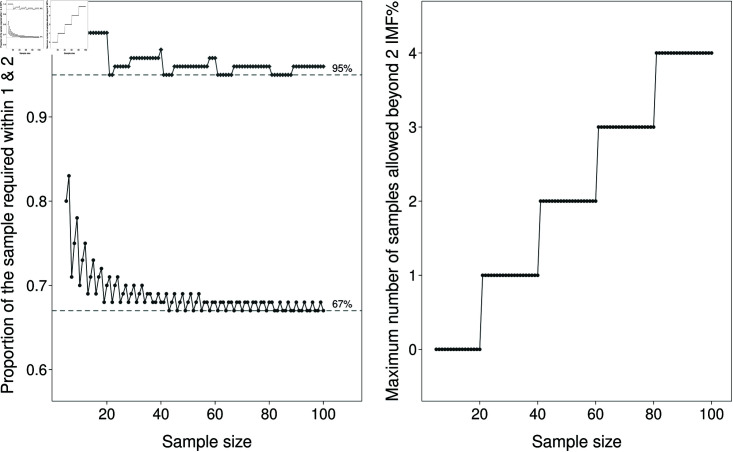
Requirements by sample size to meet accreditation rules based on thresholds. Left: The proportion of samples required to meet the rules based thresholds is inconsistent across sample size as illustrated by the see-saw effect of the proportions. This behaviour is more extreme when sample size is small but continues out to sample size 100, with proportions for some sample sizes needing to be greater than 68%. Right: The maximum number of samples allowed to fall beyond 2 IMF% is clustered into sample size increments of 10. For samples up to size 20, none of the samples may fall outside 2IMF%, then for 21–30 there can be no more than 1 sample.

As the sample size in accreditation experiments may be smaller than 100 (over ranges), the calculation of percentages will be affected by rounding values upward and subject to failure through the random presence of outliers. For example, Table 1, shows that sample sizes of less than 20 will fail accreditation if they record a single deviation greater than 2IMF%. Similarly, between sample sizes of 20 through to 50, more than one deviation in a sample beyond this boundary will also result in failed accreditation. In effect, this highlights the sensitivity of this assessment method to random outlier readings. This issue can be overcome through increasing the sample size, however, this strategy will result in substantially increased expenses that will ultimately be passed on to the sheep industry in which the technology is commercialised. Fig 3 illustrates the required proportions through to samples of size 100 that need to conform to meet the rules based requirements.

**Table 1:** Minimum number of units needed to fall within brackets to meet rules based accreditation dependant on group sample size.

Sample size	# ≤ 1IMF (%)	1IMF < # ≤ 2IMF	# > 2IMF
5	4 (80%)	1	0 (0%)
15	10 (67%)	5	0 (0%)
19	13 (68%)	6	0 (0%)
20	14 (70%)	5	1 (5%)
25	17 (68%)	7	1 (4%)
29	20 (69%)	8	1 (3.4%)
30	20 (67%)	9	1 (3.3%)
50	34 (68%)	14	2 (4%)

There are clear disadvantages associated with some sample sizes, with proportions changing based on the conversion of the discrete number of samples being taken. This aspect is highlighted by the step nature displayed in the requirements for  ±  2IMF%, and the see-saw pattern of proportions required to be  ±  1IMF%. In effect, this inconsistency will, either consciously or inadvertently, result in manufacturers gravitating towards using sample size numbers that best allow their device to pass accreditation standards, at the expense of allowing the accrediting body to gain real understanding of the device performance. For example, if a manufacturer was to change from a sample size of 15 to 19, from the additional 4 samples, 3 must be within 1 IMF%, and none can be greater than 2 IMF% (Table 1). Indeed, these inconsistencies may require manufacturers to commit to sample size before experimentation can commence. Any additional sampling would need to be built into the pre-trial protocol as an adaptive sampling framework.

### Regression approach

A regression approach is proposed to account for sampling uncertainty and alleviate issues in regards to the accreditation range. The proposed model is based on dividing the sampling range into quarters and applying a test of accuracy within each of these quarters. This provides confidence that the accreditation accuracy standards are met across each quarter of the accreditation range of IMF%. This approach also ensures the regression model is not misused by sampling predominantly in regions where the device best meets the requirements for accreditation, in effect biasing the accreditation by “data-stacking” within IMF% ranges where the technology demonstrates superior predictive performance. The regression model is as follows:


$$\begin{array}{ccc} {\text{IMF\%}}_{\text{Lab}}-{\text{IMF\%}}_{\text{Device}}=\beta_{0}+\beta_{{\text{Q}}_{2,3,4}}+\varepsilon & & \end{array}$$ (1)

In this representation:

- IMF%Lab represents the laboratory estimate of IMF.
- IMF%Device represents the device estimate of IMF.
- Then IMF%Lab - IMF%Device represents the difference between the laboratory and device estimates. This measurement is required to meet (or better) a N(0,1) distribution.
- *β*_0_ is the intercept term in the model. This represents the overall mean of the disparity between the device and the laboratory estimate of IMF%.
- *β*_Q_2,3,4__ represent the deviations from the mean disparity of the measurements in each of the 3 quarters 2-4. The first quarter is incorporated in the intercept (*β*_0_) estimate.
-  ±  represents the error term of the model which we expect to be distributed as *N*(0,*σ*^2^).

If we had near perfect correspondence between the device and the laboratory estimate of IMF%, we would expect {*β*_0_} to be approximately zero (0), and if the disparity between measures is uniform across quarters we expect {*β*_Q_2__, *β*_Q_3__, *β*_Q_4__} to also be approximately 0. Correspondingly, if these conditions are met, and the variability between the device and the laboratory met the required accuracy for accreditation, we would expect *σ* ≤ 1.

In a Bayesian approach, we need to choose a prior for the parameter estimates *β* and *σ*. In this analysis we use conjugate priors as follows:


$$p(\beta |\sigma^{2},X)∼\text{N}\left(\tilde{\beta },\sigma^{2}M^{-1}\right)$$

where *M* is a positive definite symmetric matrix, and


$$p(\sigma^{2}|X)∼\text{IG}(\alpha ,\delta )$$

where *α* , *δ* > 0. Throughout this section we will color code parameters that are assigned in the prior distribution (hyperparameters) in **red** and parameters calculated from the sample data are in standard black.

The hyperparameters $\left\{\tilde{\beta },M,\alpha ,\delta \right\}$ are used to express aspects of our expected accuracy of the device before data is collected. We can use the prior distribution to enhance diagnostics based on known factors, which is beneficial when sample size is restricted. In terms of device accreditation, sample size is restricted due to costs, which are around $30 per sample.

Based on industry discussions, it is sensible to set $\tilde{\beta }=0$, implying that on average, we expect the difference between the device and the laboratory estimate to be zero. The variance associated with $\tilde{\beta }$ is then linked to the overall variance of the residuals (*σ*^2^), we can tighten the variance by making *M* larger, and increase the variance by choosing smaller values for *M*. In our approach we treat this as a Zellner’s g-prior [5,6], with $M=g{\left(X^{T}X\right)}^{-1}$, hence manipulating the variance associated with *β* by increasing or decreasing the value of *g*. Indeed, if *g* is set to 1, the prior variance of $\tilde{\beta }$ is then equal to the estimates from a least squares procedure.

Coefficients are estimated using the standard closed form solution [7,8]. These details are provided in S1 Appendix.

The main advantage of this framework for the purpose of device accreditation is its adaptation to predict future observations from a device using the predictive posterior distribution [9–11]. As sample size will be shown to be a major issue for the rules based approach, we can mitigate the effect of the small samples with which we are required to make an assessment of the technology. The predictive posterior distribution of this model can be represented as follows [7]:


$$p(ỹ|y,X,\tilde{X})∼T_{m}\left(n+2\alpha ,\hat{\theta },\hat{\tau }\right)$$

where $\tilde{X}$ is an *m* × ( *k* + 1 )  matrix of new observations, and the mean and variance of the t-distribution are:


θ^=X~ (gXTX+XTX)−1(XTXβ^+0gXTXβ~)=X~β^(1+g) (2)

τ^=2δ+s2+(0β~−β^) ((gXTX)−1+(XTX)−1)−1(0β~−β^)n+2α (Im+X~ (gXTX+XTX)−1) (3)

**Fig 4:**
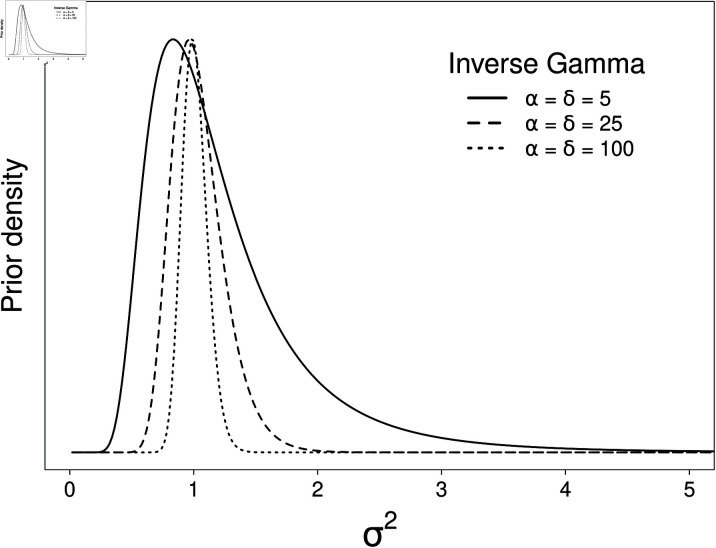
Prior density of *σ*^2^. Representation of *α* = *δ* = { 5 , 25 , 100 } . The distribution of potential values of *σ*^2^ become less variable as both *α* and *β* increase. Hence larger values of *α* and *β* have greater influence on the derived posterior distributions.

Conceptualisation of appropriate hyperparameters for the prior of *σ*^2^(α, *δ*) leads to a strong advantage of this Bayesian approach in terms of maximising the benefit of this framework when sample size is necessarily restricted. Given that the benchmark for accreditation is $\sigma^{2}\approx 1\text{IMF\%}$, it is reasonable to set this prior to a distribution with a median of around 1. Further, IMF% has a very small range in lamb (1-9%), so for developers to attempt accreditation, these devices are most likely to be providing predictions that fall within this range. This justifies setting a prior that reflects a small standard deviation around the median of 1. In this case, we can set *α* = *δ*, which will result in a prior distribution centred around 1 (Fig 4). Finally, for the prior to have any meaningful influence on our predictions of device performance we need to consider the contribution they make in and 3. For *α* to have meaningful impact on predictions, it needs to be of a magnitude that is comparable to the overall sample size *n* (). Similarly, *δ* needs to be of a magnitude that similarly impacts on the variance *τ*. As $\tilde{\beta }$ is set at 0, the other hyperparameter *g*, which is positive, will reduce the estimated mean ($\hat{\theta }$) by its role in the denominator (), however, as $\hat{\beta }$ is expected to be a value near zero, this impact is minimal over most values of *g*.

Overall, given the applied problem has expected values for $\hat{\beta }$ of near 0, we note that the value of *g* is expected to have little impact on either the value of the mean ($\hat{\mu }$), as the numerator in the equation is close to zero, hence the denominator has little effect by increasing or decreasing. Similarly, the magnitude of the variance of the t-distribution ($\hat{\Sigma }$) is largely determined by the sample size (*n*), the hyperparameters of the variance (*α* , *δ* )  and the sum of squared errors (*s*^2^). To ensure device accreditation is “data-driven”, setting the values of *α* and *δ* is the most crucial in this application, and we will determine acceptable values for these parameters in the Results section.

Although the Bayesian framework does not have the same requirements on sample size as its frequentist counterparts, in this case, larger sample sizes will still be advantageous in that it will reduce variance in posterior estimates of both coefficients and error variance [9]. This will result in less variance in the population posterior distribution, and as such, aid in ensuring devices that meet the industry standard are more likely to pass the 67% and 95% thresholds.

## Results

### Simulated data pass rate - rules approach

**Fig 5:**
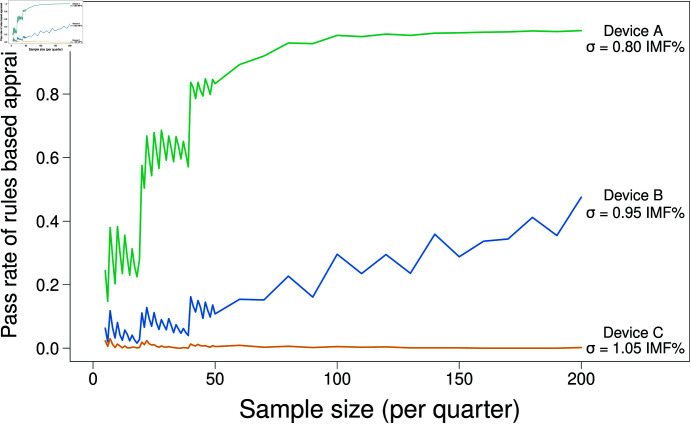
Pass rate of all quarters for 1,000 simulations from a N(0,0.80^2^), N(0, 0.95^2^) and N(0,1.05^2^) distribution.

In the rules based assessment, data were allocated to one of 4 quarters. For a simulation to pass, it must meet the accreditation standards in each of these quarters. Fig 5 shows the pass rate of the 1000 simulations for 3 of the theoretical devices (Devices A, B & C), with Device D excluded from the graph as it rarely passed the rules based requirements.

Fig 5 (**green** line) shows that even the most accurate data (Device A) which performs well within the required performance, a N(0, 0.80^2^), rarely passes all 4 quarters simultaneously until the sample size is quite large. In this case, a sample size of around 100 per quarter (Total sample size 400) was required to approach the 100% pass rate, while sample sizes of around 50 per quarter were required to achieve an 80% pass rate. Device B (**blue** line), which is just within accreditation limits, a N(0, 0.95^2^), does quite poorly, with a pass rate of all 4 quarters reaching around 50% at a sample size of 200 per quarter (800 total). This result is highly undesirable to the industry as this device is performing satisfactorily and its accreditation is desirable.

For the two devices that meet accreditation standards (Device A & B), the rounding effect of the rules-based approach is evident, particularly with sample sizes of less than 50 (per quarter). In this case, pass rates changed by 10-15% through the addition of an extra sample. This pattern continued out to sample size 200, although the effect dampened as the sample size increased.

Device C, which does not meet accreditation standards, performing at (N(0,1.05^2^) accuracy, seldom passed, even with large sample size ( ≈  0% pass rate), and passed at less than 5% rate in small samples. This is illustrated in Fig 5 with the **orange** line.

Fig 5 highlights the main problem with the rules based approach, in that devices that should pass, e.g. Device B with a N(0,0.95) distribution of errors based on laboratory estimates, does not even achieve a 60% pass rate when sample size is 200 per quarter. This apparent failure of the rules based approach is due to the limitations in the number of samples that can be obtained.

**Fig 6:**
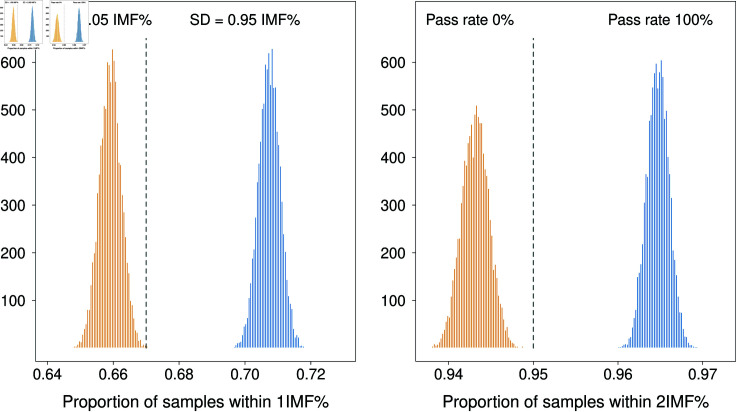
Results of 10,000 simulations from a N(0,0.95^2^) and N(0.1.05^2^) with sample size 20,000. The overall pass rate of the simulated device is 100% for the N(0,0.95^2^), and none of the simulations passed for the device represented as having a standard deviation of 1.05IMF% units.

Fig 6 shows the distribution of 10,000 simulations of sample size 20,000 from the device that should pass accreditation, and the device that is slightly more variable than allowed. We can see that for a large sample (20,000), a device that deviates from laboratory estimates with mean 0 and standard deviation 0.95 (**blue** histograms) will pass both the requirement of  ≥  67% of measurements within  ±  1 IMF% of laboratory estimate and  ≥  95% of measurements within 2 IMF% for 100% of simulations. Whereas none of the simulations for a device with deviations equivalent to N(0, 1.05^2^), the **orange** histograms, were able to pass both requirements at the 1 IMF% and 2 IMF% thresholds. The rules based approach would require very large samples to satisfactorily determine adherence to the industry requirements, and samples of this magnitude are prohibitively expensive for the industry.

### Simulated data pass rate - regression approach

The same set of simulated data was used to assess the performance of the regression based approach. For each simulation the following steps were performed:

- Divide the data range of device measurements into quarters.
- Allocate each measurement to it’s appropriate quarter.
- Calculate $y={\text{IMF\%}}_{\text{Lab}}-{\text{IMF\%}}_{\text{Device}}$.
- For each sample size required (*n*), use the first *n* samples of the simulation.
- Simulate 50,000 values from the predictive posterior of the regression model.
- The hyperparameter *g* for the prior mean standard deviation is set at 1.
- The hyperparameters *α* and *δ* were assessed at 4 values {sample size in smallest quarter, 0.5n, n, 2n}

**Fig 7:**
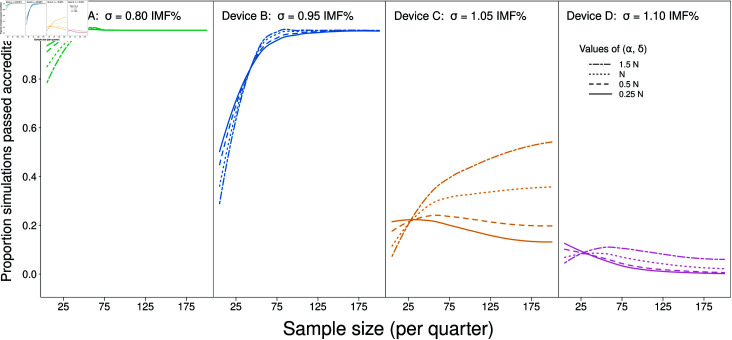
Pass rate of all quarters for 1,000 simulations from theoretical devices using the regression approach. The theoretical devices follow a) N(0,0.80^2^), b) N(0, 0.95^2^), c) N(0,1.05^2^) and d) N(0,1.10^2^) distributions. The drawn sample size of the posterior predictive distribution is 50,000. Varying values of the hyperparameters *α* = *δ* are represented.

The pass rate of the devices (A, B, C & D) under the four variations of prior hyperparameters (*α*, *δ*) is illustrated in Fig 7. This demonstrates that setting *α* = *δ* = 0 . 25*n* was most beneficial in terms of device accreditation for all 4 devices when the sample size was low (less than  ≈  120 total), and this behaviour switches as sample size increased.

**Fig 8:**
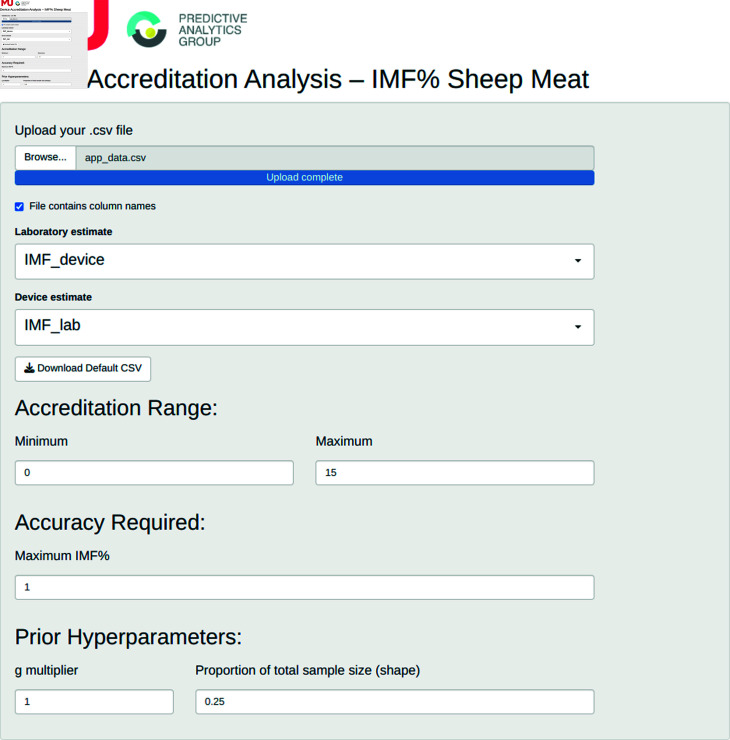
App Data Upload and Parameter Setting. A csv file containing columns with the laboratory and device estimates is required. Varying values of the hyperparameters *g* and *α* = *δ* can be trialled.

In all scenarios, Device A, which was well within accreditation limits, a N(0,0.8), passed 100% of the time when sample size exceeded 30 per quarter (Fig 7, **green** lines), and Device B, which just satisfied the requirements, being N(0,0.95), passed accreditation in excess of 80% of the time using the same prior distribution (**blue** lines). Using this methodology, the N(0,0.95) device passed near 100% of the time with the strongest tested prior at sample size 75 per quarter, and at around 125 per quarter when using the weakest tested prior (Fig 7). In terms of ensuring that devices that are not meeting standards do not pass, choosing (*α*, *δ*) to be between 0 . 25*n* through to 0 . 5*n* minimised false pass-results. This was evident with Device C, that is just outside the accreditation standards, passing less than 20% of the time at low sample size, and decreasing in pass rate as sample size increased above 30 per quarter (Fig 7, **orange** lines). The hyperparameters in this prior distribution scheme had negligible effect when the device was well outside the accreditation standards, in this case N(0,1.1), with all instances obtaining accreditation in less than 10% of the simulations (Fig 7, **pink** lines).

### Practical implementation

Assessment of devices seeking accreditation is conducted using this statistical framework within an R Shiny App [12].

The first stage of the process is to upload the data from the device accreditation experiments. This data requires a column of data that provides the laboratory estimate of IMF and a corresponding column with the measurements obtained by the device seeking accreditation (See Fig 8). The uploaded data is not stored in any database. As a result, users will need to re-upload their data in any additional sessions.

**Fig 9:**
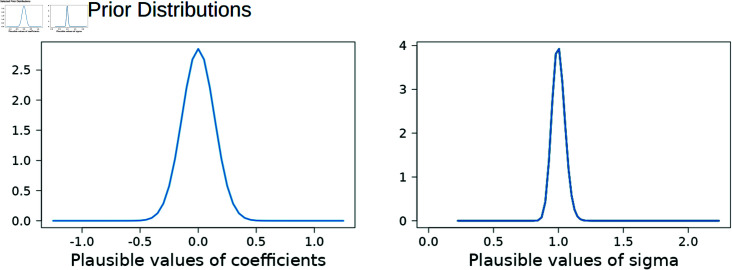
Prior distributions associated with selected hyperparameters. The user can visualise the density of the prior distribution associated with the chosen hyperparameters *g* and *α* = *δ*.

The prior distribution hyperparameters can also be set in this panel, with *g* restricted to the range (0.25, 5.00) and *α* = *δ* restricted to the range (0.05 x *N*, *N*) where *N* is the total sample size. These range limitations were determined from the results of the simulation study and industry consultation. The plausible values under the prior distribution selected are illustrated graphically as distribution plots (Fig 9).

**Fig 10:**
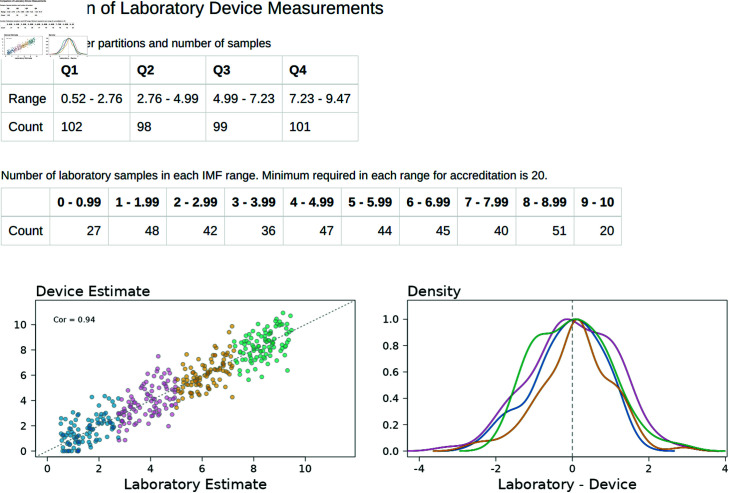
Initial assessment of a device. In the Shiny App, data are uploaded and allocated to quarter ranges.

Once data is uploaded, the next step on this tab is to visually assess the performance of the supplied device, and assess whether the minimum sampling density is met. The initial “Data Summary” tab is shown in Fig 10. In this example, the assessor would determine that the device cannot achieve accreditation in the final quarter (IMF% range 6.63–8.38) as there are only 9 samples, which is not enough to meet the minimum sample requirements. Furthermore, visual inspection of the first quarter (1.35–3.11) indicates it may not pass accreditation as the bulk of the 397 samples are located above the 1:1 line, implying excessive overestimation of IMF% in this range. This idea is reinforced by the density plot of residuals (blue line), with the mean residual of this range being approximately  − 1 as the bulk of the density fell in the negative range.

From Fig 10, we note that the 2nd and 3rd quarter ranges both have sufficient samples, and a visual inspection indicates that the prediction accuracy may be adequate to pass accreditation. The 2nd quarter (3.11–4.87), indicated by pink in the graphs, appears to be uniformly scattered around the 1:1 line, and the density plot (pink line) of its residuals is centered near 0 and reasonably symmetric. The 3rd quarter (orange color coded), which ranges between (4.87–6.63), is slightly more variable, with a residual density centered slightly positive with some negative skewness. At this point, the range included for accreditation can be modified using the control panel, excluding data until the more sparsely sampled upper quarter meets the minimum sampling requirement of n=20. Excluded data will be recoloured in grey on this screen.

**Fig 11:**
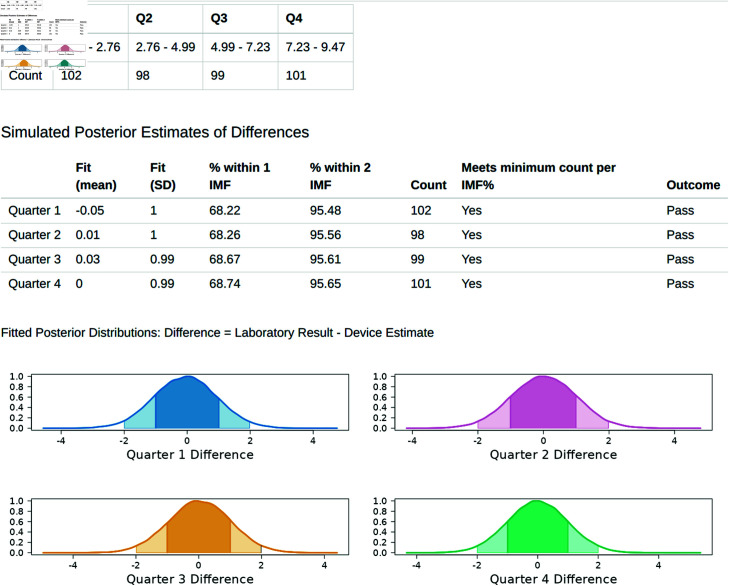
Regression Tab. The posterior distributions and sample size rules determine the pass/fail of a device over each quarter.

The “Regression Analysis” tab contains the results of the fitted model (Fig 11). This tab shows a table of the simulated posterior estimates of the differences (Fitted Mean & Standard Deviation), the percentage of these estimates that fall within ±1 IMF% and ±2 IMF% of the laboratory value, and the accreditation outcome for each quarter of the data range. Within each quarter a successful accreditation outcome is based upon greater than 67% of these estimates falling within ±1 IMF% and greater than 95% within ±2 IMF% of the laboratory value, as well as the number of observations (Count) being greater than 19 within that quarter. Lastly, 4 graphs are shown representing the fitted posterior distributions for each quarter, with the shaded regions representing that section of the distribution falling within ±1 IMF% and ±2 IMF% of the laboratory value. In this example, the accreditation standards are met across all quarters, but quarter 4 fails on the basis of too few sample numbers within the range, and quarter 3 fails on the basis of the IMF% range for 5-5.99 having too few samples.

**Fig 12:**
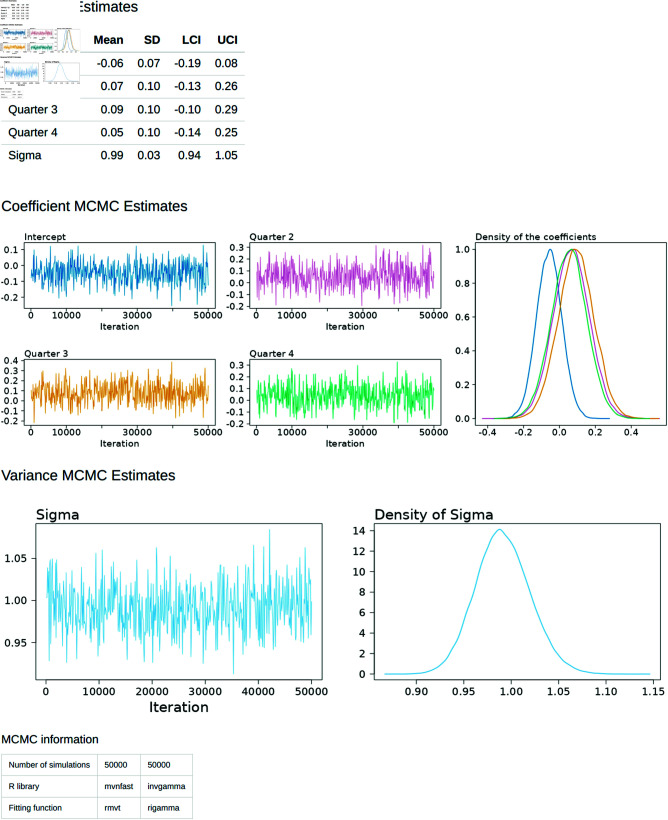
Diagnostics Tab. The model estimates are assessed visually and in terms of Credible Intervals.

The “Regression Diagnostics” tab (Fig 12) shows a table consisting of the coefficient estimates of the regression model along with their lower (LCI) and upper (UCI) 95% confidence intervals. The Monte Carlo Markov Chain (MCMC) trace plots are provided to demonstrate stability of the coefficient estimates, with the accompanying density plots demonstrating plausible values for the regression coefficients (*β*) and the standard devistion (*σ*).

**Fig 13:**
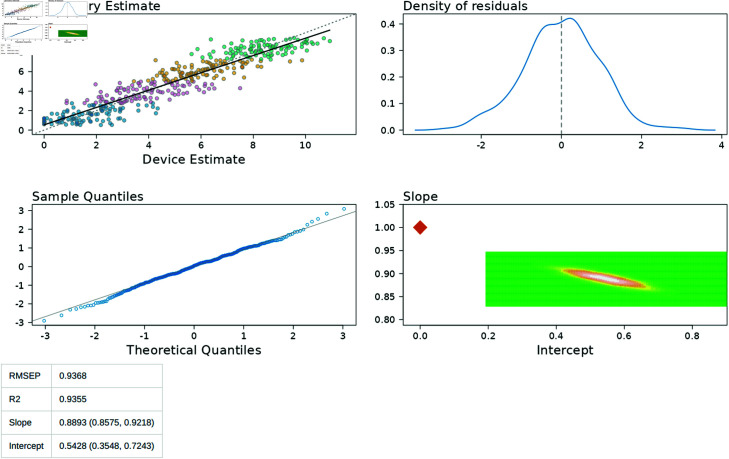
Standard Regression Tab. Standard reporting measures for the accreditation report.

The “Standard Regression” tab (Fig 13) provides additional reporting statistics required for the accreditation report. This includes a scatter plot of the laboratory measured IMF% (x-axis) versus the device estimated IMF% (y-axis), and a fitted regression line through this data. The intercept and slope of this regression are reported, along with the root mean square error of the prediction (RMSEP) and R2 values. The points in this scatter plot are colour coded to represent the allocation of data to quarters. Additional diagnostic graphs are provided that relate to the scatter plot. These include the distribution of residuals, a QQ plot, and a graphical representation of the plausible intercept and slope values.

Two other tabs included provide “Getting Started” information for new users, and “Statistical Methodology” information for those seeking to describe and publish their accreditation analysis. This enables untrained users to access and utilise the analysis package.

## Discussion

Aligning with our initial hypothesis, the regression method required less samples to arrive at robust estimates of accreditation accuracy. This was well demonstrated by the relationship between the probability of passing accreditation versus the number of samples represented per IMF% increment, and became particularly evident when the simulated error of the accrediting technology was approaching the threshold of 1 IMF%. For a device with simulated error of 0.95, when using the rules-based method, approximately 100 samples were required to achieve a 0.8 probability of passing accreditation. In contrast the regression method required only 25 samples to achieve a similar level of confidence. This provides several advantages to industry. Firstly, the method improves the robustness of the accreditation outcome with fewer samples required. This will give industry confidence in the accuracy of the measurement technologies underpinning their trading. This will enhance supply-chain transparency and trust, facilitating trade and the accurate transfer of value between supply-chain participants. Secondly, the method limits the dependence of the outcome on sample size. Given the $30 per sample cost of undertaking IMF% analyses, this represents an important cost-saving, an expense that would otherwise be passed back to industry through greater expense for the device sold by the technology provider. Furthermore, greater costs can also deter technology providers from seeking accreditation resulting in fewer technology solutions available to industry. Thirdly, the reduced requirement for samples will increase the speed of accreditation. During accreditation, the most difficult animals to source are those at the extremes of the population. These can be very difficult to acquire in normal commercial populations, so ultimately the reduced sample numbers will limit the number of days spent sampling in an attempt to find these outlier animals. The end result is that industry are provided with cheaper measurement technologies, with robustly proven accuracy that are accredited across a diverse IMF% range.

The other advantage of the regression method is in the scenario where a technology company has a device that only just meets the accreditation accuracy, for example a N(0, 0.99). In this case the rules-based approach will likely never exceed 50% probability of passing, no matter how high the sample numbers become. This represents a test that is too conservative, unfairly excluding this technology from commercialisation, and restricting the device options that are available to industry. In contrast, the regression approach creates an avenue to accreditation for these less accurate technologies that would be achievable with sufficient sample numbers. An alternative solution would be to make the accreditation standards more lenient, but this would erode industry confidence in the reliability of any accredited technology.

Intramuscular fat percentage is also a key determinant of beef eating quality, with this trait released within the Australian Beef Industry trading language in 2022 [13]. The analytical approach described in this paper is entirely applicable to devices seeking accreditation to measure IMF% in beef, although some alterations are recommended. Firstly there is the issue of data range. Beef IMF% values exceed those of lamb and therefore the accuracy must be assessed across a greater range of IMF% values. To ensure adequate resolution across this range, more tests are required. Secondly, there is a need for alignment with the existing visual marble score systems currently used by the beef industry. The AUS-MEAT marble scores extend from 0 (low marbling) to 9 (high marbling), while for the Meat Standards Australia (MSA) marble score system the visual range is similar, although units are expressed between 100 and 1190 MSA marble scores. In both cases a change of 1 AUS-MEAT marble score, or 100 MSA marble scores is equivalent to approximately 3 IMF% units. Therefore to establish some level of compatibility we would recommend imposing tests of accuracy for beef IMF% in quarters of the data range up to 12 IMF% units, and then impose an additional test for every 3 IMF% beyond the range of 12. In this way, industry are given confidence that accredited technologies maintain their accuracy across an industry relevant range, and that this accuracy is maintained within segments equivalent to their existing visual grading standards.

A key advantage of Bayesian methodologies is the use of priors to inform the model fitting process. However, in this case, it was important that the priors did not unduly influence the outcome of the accreditation analysis. Manipulating the priors in order to successfully meet the accreditation error tolerance would erode confidence within industry. Considering Fig 4, choosing hyperparameters for *α* and *δ* that range between a quarter and half the total sample size, intuitively, will provide a realistic prior expectation of a device participating in the accreditation procedure. As we note from this graph, choosing hyperparameters with a magnitude of 25 represent a prior belief that the value of *σ*^2^ is between approximately 0.5 and 1.75, and increasing the magnitude of the hyperparameters further restricts this to values around 1. When the variation in the sampled data exceeding the anticipated value of this prior, terms such as the sum of squared errors (*s*^2^) become larger in magnitude due to increased variability, and therefore increase the variation associated with the predictive posterior. This behaviour results in a posterior that is closer to the data estimates, rather than being dominated by the prior expectations, and as such, having seemingly informative priors is not unduly influencing the outcome.

A crucial facet supporting the commercial use of the regression method has been on-line access to the analysis App. The processing speed, ease of use, informative diagnostics, and methodological description have enabled seamless access and usability. Crucially, this has enabled technology companies that are developing devices to track performance during the calibration phase of development, providing confidence to cover the expense of seeking accreditation. Furthermore, this has provided the accrediting bodies in Australia, including AUS-MEAT and the Australian Meat Industries Language and Standards Committee, with a standardised output that enables them to scrutinise the statistical compliance to the accreditation standards of any device submissions. This includes clear interpretive statements of whether a technology has passed or failed accreditation. Users with only a broad understanding of statistics have the capacity to use and interpret the outputs from the App, eliminating the need to learn complex statistical code. These Apps for both lamb and beef IMF% accreditation can be accessed via the following extensions:

Link to LAMB App:

https://accreditationapps.shinyapps.io/SheepMeatIMFPercentVersion5/

Link to BEEF App:

https://accreditationapps.shinyapps.io/Beef_IMF_App/

## Conclusion

In conclusion, we have developed a Bayesian regression method that tests the accuracy of predictions made by technologies relative to a set of accreditation standards for measuring IMF% in lamb. This method requires less samples than a simple rules based approach to arrive at robust estimates of accreditation accuracy, therefore reducing costs and increasing confidence within industry. This method has been adopted by industry for analysis of accreditation data. We have also highlighted the applicability of this method to beef IMF%, with some alterations required to handle the greater IMF% range expected in this species. Lastly, to support the commercial use of this method we have developed an on-line analysis App, which commercial users can freely access to test the accuracy of their own technologies.

The R code used to simulate this data and model the outcomes is available at doi: 10.60867/00000024.

### S1 Appendix.

**Posterior distribution of regression coefficients**

Using the conjugate priors outlined in this paper, the conditional posterior density of *β* is then a Normal distribution as follows:


$$p(\beta |\sigma^{2},X,y)∼N_{k+1}\left({\left(M+X^{T}X\right)}^{-1}(X^{T}X\hat{\beta }+M\tilde{\beta }),\sigma^{2}{\left(M+X^{T}X\right)}^{-1}\right)$$

The marginal posterior density of *σ* is an inverse gamma distribution:


$$p(\sigma^{2}|\beta ,y,X)∼\text{IG}\left(\frac{n}{2}+\alpha ,\delta +\frac{s^{2}}{2}+\frac{{\left(\tilde{\beta }-\hat{\beta }\right)}^{T}{\left(M^{-1}+{\left(X^{T}X\right)}^{-1}\right)}^{-1}(\tilde{\beta }-\hat{\beta })}{2}\right)$$

For simplification we can integrate the conditional posterior distribution of *β* over the marginal posterior for *σ*^2^ which will yield a marginal posterior distribution for *β* that is a multivariate t distribution (Strickland et al.). The advantage of this framework is that the model now has a closed form solution, which will speed up computation over the traditional Markov Chain Monte Carlo sampling of the posterior distribution.


$$p(\beta |X,y)∼T_{k+1}\left(n+2\alpha ,\hat{\mu },\hat{\Sigma }\right)$$

where


$$\hat{\mu }={\left(M+X^{T}X\right)}^{-1}\left((X^{T}X)\hat{\beta }+M\tilde{\beta }\right)$$

$$\hat{\Sigma }=\frac{2\delta +s^{2}+(\tilde{\beta }-\hat{\beta }){\left(M^{-1}+{\left(X^{T}X\right)}^{-1}\right)}^{-1}(\tilde{\beta }-\hat{\beta })}{n+2\alpha }{\left(M+X^{T}X\right)}^{-1}$$
